# A frameshift variant in *PKP2* can be associated with a complex phenotype in sudden cardiac death: a case report

**DOI:** 10.1093/ehjcr/ytag067

**Published:** 2026-01-29

**Authors:** Gustav A Davidsson, Gudny A Arnadottir, Gardar Sveinbjornsson, Helga Ottarsdottir, David O Arnar

**Affiliations:** Cardiovascular Services, Landspitali—the National University Hospital of Iceland, Hringbraut, 101, Reykjavik, Iceland; Clinical Sequencing, deCODE genetics/Amgen, Sturlugata 8, 101 Reykjavik, Iceland; Clinical Sequencing, deCODE genetics/Amgen, Sturlugata 8, 101 Reykjavik, Iceland; Children´s Hospital, Landspitali—the National University Hospital of Iceland, Hringbraut, 101 Reykjavik, Iceland; Cardiovascular Services, Landspitali—the National University Hospital of Iceland, Hringbraut, 101, Reykjavik, Iceland; Clinical Sequencing, deCODE genetics/Amgen, Sturlugata 8, 101 Reykjavik, Iceland; Faculty of Medicine, The University of Iceland, Vatnsmyrarvegur 16, 101 Reykjavik, Iceland

**Keywords:** Sudden cardiac death, arrhythmogenic cardiomyopathy, whole genome sequencing, QT prolongation, case report

Learning points

- Whole-genome sequencing can be very useful in evaluation of cardiac arrest victims and family members when the underlying cause is uncertain.
- Arrhythmogenic cardiomyopathy can be associated with a complex phenotype, including a prolonged QTc interval.
- Abnormal repolarization in arrhythmogenic cardiomyopathy may be an indicator of an increased risk of life-threatening arrhythmias.

## Introduction

Cardiac arrest in a young individual is among the most challenging scenarios in clinical medicine. Finding the cause for such an event is of utmost importance so the correct approach to evaluate the individual and family members can be chosen.^1^

Plakophilin-2, found within desmosomes in cardiomyocytes, is encoded by the *PKP2* gene, and defects in this gene have been associated with arrhythmogenic cardiomyopathy (ACM) and sudden cardiac death (SCD).^2^ Pathogenic heterozygous variants in *PKP2* are the most frequently identified genetic cause of ACM although reduced penetrance and variable expressivity are reported, with wide intra-familial variability.^3^

We describe a complex phenotype, ACM with a prolonged corrected QT (QTc) interval, associated with a likely pathogenic frameshift variant in the *PKP2* gene in a patient successfully resuscitated from cardiac arrest. These observations were further augmented by findings from the deCODE genetics database, with genetic information for a large fraction of the Icelandic population,^4^ and the extensive University Hospital electrocardiogram (EKG) database.^5,6^

## Summary figure

**Figure:**
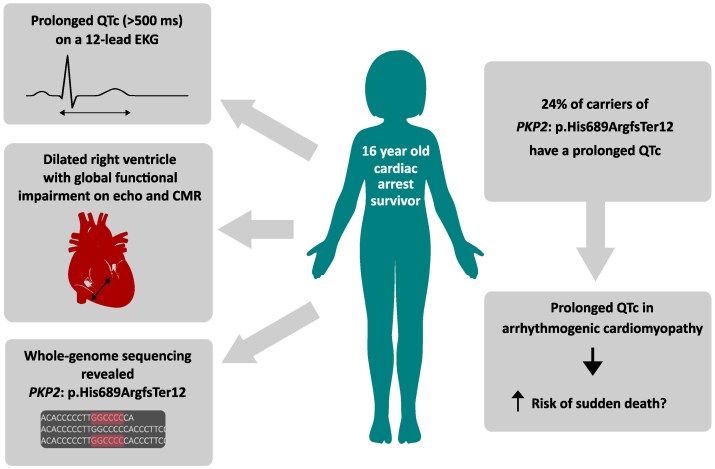


## Case presentation

**Figure 1:**
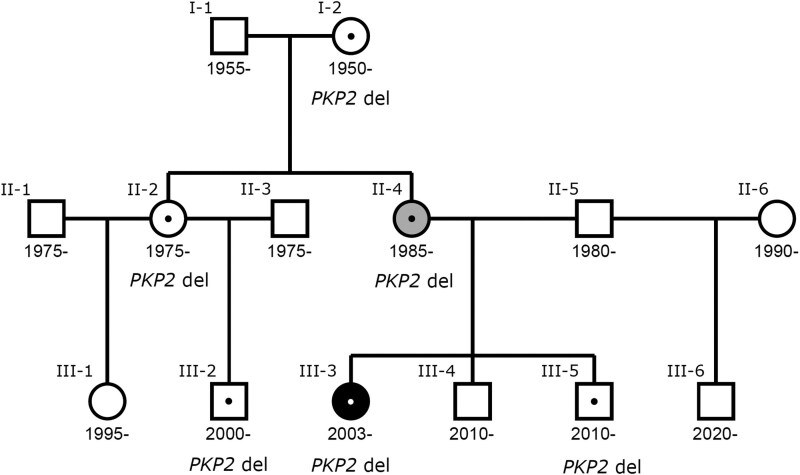
A family tree showing family members with the *PKP2* variant (black dot) and ACM phenotype (proband in black and mother in grey). This shows variable disease penetrance in the affected family members.

The proband was a 16-year-old female (*Figure 1*, III-3) who suddenly lost consciousness, and became unresponsive after intense training. A witness immediately began cardiopulmonary resuscitation (CPR), called emergency services and she was successfully defibrillated from ventricular fibrillation by the emergency personnel from the ambulance. She was previously healthy, had no know cardiovascular disease and no family history of SCD.

**Figure 2:**
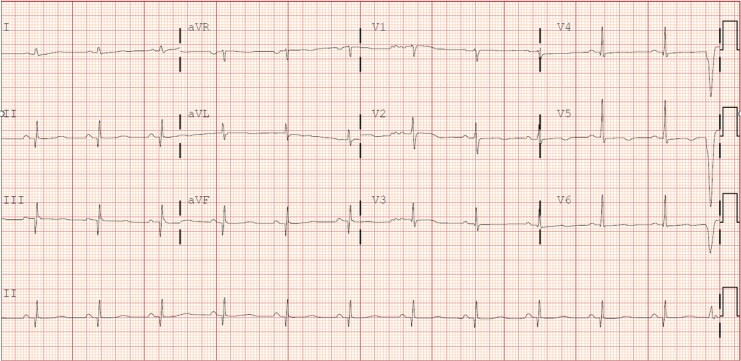
The first EKG, taken in the emergency room, of the proband showing a prolonged QTc of 532 ms. This was initially suggestive of the long QT syndrome.

Cardiovascular exam was normal, but the initial EKG taken in the emergency room revealed a considerably prolonged QTc interval of 532 ms (normal <460 ms) (*Figure 2*). A quick bedside echocardiogram showed a mildly depressed left ventricular (LV) function and a clearly enlarged right ventricle (RV). A pulmonary embolism was ruled out by a computed tomogram. The patient woke up a few hours later, completely neurologically intact, most likely due to early bystander CPR and prompt defibrillation.

The QTc was prolonged, the RV was dilated and the initial differential diagnosis suggested long QT syndrome (LQTS), ACM, or possibly even both. The LQTS is an inherited cardiac repolarization disorder that can result in SCD.^7^

**Figure 3:**
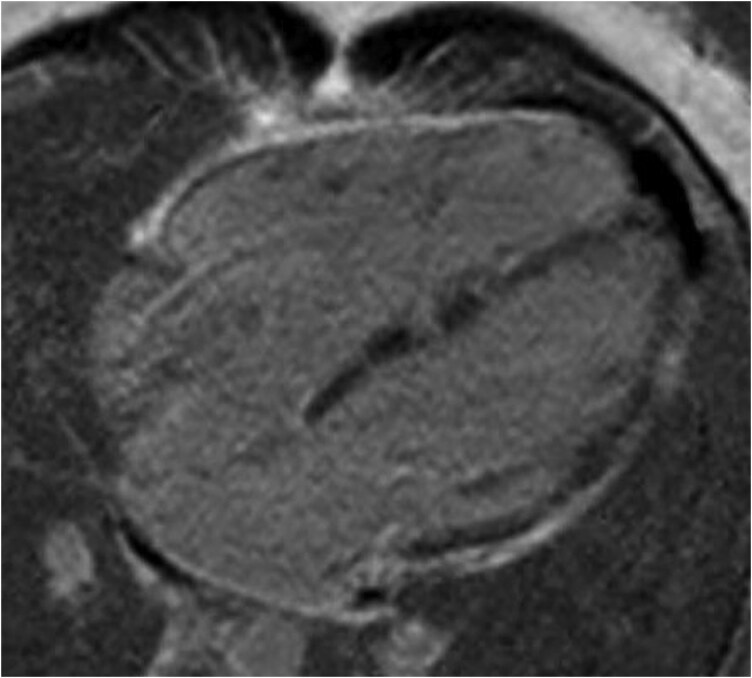
A cardiac MRI showing an enlarged right ventricle with an end-diastolic-volume-index of 151 ml/m^2^ (upper limit of normal 87 ml/m^2^) and a calculated RVEF of 30%. There was late gadolinium enhancement in the right and left ventricular walls, suggesting fibrofatty infiltration. The sequence shown is delayed enhancement inversion recovery T1. This figure shows a clear suggestion of arrhythmogenic cardiomyopathy.

A cardiac magnetic resonance image (MRI) was performed which showed an enlarged and hypodynamic RV with an end-diastolic-volume-index of 151 ml/m^2^ (normal: <87 ml/m^2^) and a RVEF of 30%. The left ventricle was enlarged, with LD end diastolic volume index of 108 ml/m^2^. CMR derived EF was 42% and there was globally depressed LV function. There was patchy delayed gadolinium enhancement in the right and left ventricular walls (septum, proximal anterior wall and lateral wall of LV and freewall of RV), suggesting fibrofatty infiltration. These findings were highly indicative of biventricular ACM (*Figure 3*).

Whole-genome sequencing was performed on DNA from the proband, her parents, and three younger brothers. A frameshift variant in *PKP2*: NM_001005242.3:c.2066_2071delinsG/p.(His689ArgfsTer12) was detected in the proband (*Figure 1*, III-3), her mother (*Figure 1*, II-4), and one of her brothers (*Figure 1*, III-5). This variant fulfils criteria as likely pathogenic. Subsequently, whole-genome sequencing was expanded to other close relatives, revealing three additional carriers (*Figure 1*, I-2, II-2, and III-2). Importantly, whole-genome sequencing did not yield any other rare coding variants in genes previously associated with LQTS. This, along with the MRI findings, confirmed the diagnosis of ACM.^8^

All relatives carrying the variant had a thorough cardiac evaluation, including cardiac MRI, but except one (II-4) had a normal phenotype, the exception being the proband’s mother, who had a mild ACM but a normal QTc.

The proband was treated with a beta blocker and an implantable cardioverter defibrillator (ICD). She received two appropriate shocks from her ICD eight months later. ICD interrogation revealed monomorphic ventricular tachycardia, which is more consistent with an arrhythmia due to ACM rather than LQTS, which frequently results in a polymorphic VT termed *torsade de pointes*. Her beta blocker dose was increased, and she has been arrhythmia free for more than three years. The QTc has remained prolonged and measured 593 ms 4 years after the cardiac arrest.

The relatives with a positive genotype were all treated with beta blockers and offered advice regarding exercise restriction.

## Discussion

This case of a young woman resuscitated from cardiac arrest demonstrated the unusual initial presentation of two distinct phenotypes, LQTS and ACM. Subsequent evaluation with cardiac MRI revealed findings consistent with ACM and whole-genome sequencing showed that the patient had a sequence variant that associates with ACM with the observed QTc prolongation most likely a secondary phenomenon.

**Figure 4:**
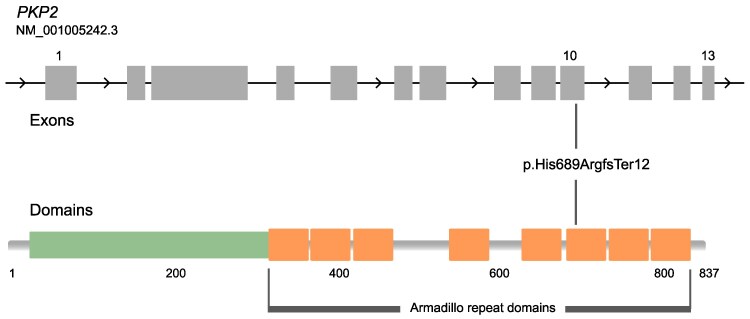
A schematic representation of the exon and domain composition of *PKP2*. The p.(His689ArgfsTer12) variant in *PKP2* is located in exon 10, within the sixth armadillo repeat domain of the plakophilin-2 protein. This schematic illustrates the location of the p.(His689ArgfsTer12) variant relative to *PKP2,* which can predict its effect on the protein product. The upper panel shows that the variant is located in exon 10 out of 13 exons, i.e. not limited to the last exon, and is thereby expected to lead to nonsense mediated decay rather than to produce an end-truncated protein product. The lower panel shows that the variant would remove amino acids 701–837, or roughly 16% of the protein which surpasses the 10% threshold used as a prediction of nonsense mediated decay over end-truncation.

The p.(His689ArgfsTer12) variant consists of a deletion of six nucleotides and insertion of one nucleotide, resulting in a shift in the reading frame and introduction of a premature stop codon eight amino acids downstream of the variant location (*Figure 4*). This variant is reported to cause ACM, submitted eight times as a pathogenic variant on ClinVar (ClinVar ID: 689321).

A considerable fraction of the Icelandic population has been either whole-genome sequenced (72 000) or chip-genotyped (173 000) by deCODE genetics, or 19% and 46% of 380 000 inhabitants, respectively. This, together with an extensive genealogy database, allows for accurate assessment of the frequency of sequence variants in the population. The *PKP2* p.(His689ArgfsTer12) variant is detected in heterozygous state in 319 chip-genotyped Icelanders, 168 of whom were also whole-genome sequenced [minor allele frequency (MAF) of 0.09%]. The variant is carried by 112 individuals of European descent among 589 822 sequenced European exomes on gnomAD (MAF 0.01%), indicating an increased frequency in Iceland likely due to a founder effect.

The University Hospital EKG database includes all EKG obtained and digitally stored since 1998 and includes 601 671 EKGs from 114 719 individuals. We recently used this data to search for variants that associate with the QTc and this same variant in *PKP2* was found to cause a mean QTc prolongation of 11.4 ms (*P* = 2.1 × 10^−8^).^6^ For this current case report we found that a total of 178 of the 319 *PKP2* variant carriers in the deCODE genetic database had an available EKG and 47 (26.4%) had an abnormally prolonged QTc (≥460 ms) (*P* = 0.00067, compared to non-carriers). Whether the observed QTc prolongation is due to effects on the action potential duration or secondary to slow conduction through diseased myocardium and subsequent abnormal repolarization is not clear. The latter seems more plausible, but the presence of this variant likely explains both abnormal findings in this case.

ACM has previously been associated with repolarization abnormalities.^9^ In that study that ACM was associated with abnormal dispersion of ventricular repolarization, and this might be a predictor of increased risk of mortality. Interestingly, in the family presented here, the proband was the only one to have a prolonged QTc interval and a cardiac arrest among the *PKP2* carriers.

## Conclusion

As shown by the data from the deCODE genetics and the University hospital EKG databases, a prolonged QTc interval is present in up to a fourth of individuals with this *PKP2* variant, making this finding clinically relevant. It has been suggested that repolarization abnormalities in ACM could be a risk marker for mortality. Making the distinction between ACM and LQTS is important in terms of overall risk stratification, management and appropriate family screening.

Determining the aetiology of cardiac arrest can be challenging, but as demonstrated by this report, next generation sequencing could play an increasing role in better understanding of complex arrhythmia phenotypes and causes of SCD.

## Lead author biography


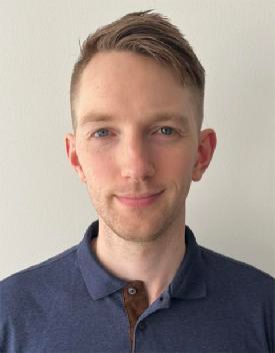


Dr. Gustav A. Davidsson obtained his medical degree from the University of Iceland in Reykjavik, Iceland. He was a trainee in internal medicine at Landspitali - The National University Hospital of Iceland in Reykjavik, Iceland and is about to begin a cardiology training position at Sahlgrenska University Hospital in Gothenburg, Sweden. His areas of interest include general cardiology, cardiac interventions and cardiac electrophysiology. Gustav was a finalist in the competition for the best case-report at the European Society of Cardiology Congress in London in 2024.

## Supplementary Material

ytag067_Supplementary_Data

## Data Availability

The data presented in this article will be shared upon reasonable request to the corresponding author.
